# Multi-omic atlas of the parahippocampal gyrus in Alzheimer’s disease

**DOI:** 10.1038/s41597-023-02507-2

**Published:** 2023-09-08

**Authors:** Claire Coleman, Minghui Wang, Erming Wang, Courtney Micallef, Zhiping Shao, James M. Vicari, Yuxin Li, Kaiwen Yu, Dongming Cai, Junmin Peng, Vahram Haroutunian, John F. Fullard, Jaroslav Bendl, Bin Zhang, Panos Roussos

**Affiliations:** 1 Department of Genetics and Genomic Sciences, Icahn School of Medicine at Mount Sinai, New York, NY 10029 USA; 2 Department of Psychiatry, Icahn School of Medicine at Mount Sinai, New York, NY 10029 USA; 3 Center for Disease Neurogenomics, Icahn School of Medicine at Mount Sinai, New York, NY 10029 USA; 4 Icahn Institute of Genomics, Icahn School of Medicine at Mount Sinai, New York, NY 10029 USA; 5 Friedman Brain Institute, Icahn School of Medicine at Mount Sinai, New York, NY 10029 USA; 6 Mount Sinai Center for Transformative Disease Modeling, Icahn School of Medicine at Mount Sinai, New York, NY 10029 USA; 7 Department of Structural Biology, St. Jude Children’s Research Hospital, Memphis, TN 38105 USA; 8 Department of Developmental Neurobiology, St. Jude Children’s Research Hospital, Memphis, TN 38105 USA; 9 Center for Proteomics and Metabolomics, St. Jude Children’s Research Hospital, Memphis, TN 38105 USA; 10 Department of Neurology, Icahn School of Medicine at Mount Sinai, New York, NY 10029 USA; 11 James J Peters VA Medical Center, Research & Development, Bronx, NY 10468 USA; 12 Alzheimer Disease Research Center, Icahn School of Medicine at Mount Sinai, New York, NY 10029 USA; 13 Nash Family Department of Neuroscience, Icahn School of Medicine at Mount Sinai, New York, NY 10029 USA

**Keywords:** Diseases, Neurological disorders, Computational biology and bioinformatics

## Abstract

Alzheimer’s disease (AD) is the most common form of dementia worldwide, with a projection of 151 million cases by 2050. Previous genetic studies have identified three main genes associated with early-onset familial Alzheimer’s disease, however this subtype accounts for less than 5% of total cases. Next-generation sequencing has been well established and holds great promise to assist in the development of novel therapeutics as well as biomarkers to prevent or slow the progression of this devastating disease. Here we present a public resource of functional genomic data from the parahippocampal gyrus of 201 postmortem control, mild cognitively impaired (MCI) and AD individuals from the Mount Sinai brain bank, of which whole-genome sequencing (WGS), and bulk RNA sequencing (RNA-seq) were previously published. The genomic data include bulk proteomics and DNA methylation, as well as cell-type-specific RNA-seq and assay for transposase-accessible chromatin with high-throughput sequencing (ATAC-seq) data. We have performed extensive preprocessing and quality control, allowing the research community to access and utilize this public resource available on the Synapse platform at 10.7303/syn51180043.2.

## Background & Summary

Alzheimer’s disease (AD) is the most common form of dementia worldwide, with a projection of 151 million cases by 2050, owing in part to our aging global population^1^. Since Dr. Alois Alzheimers’ seminal discovery over a century ago, when he described “A peculiar severe disease process of the cerebral cortex”, there have been a plethora of theories. However, clinical trials of disease-modifying treatments have been largely unsuccessful^2^. While forgetfulness and the loss of memory were always considered the first disease symptoms, spatial navigation and orientation deficits have been increasingly shown in preclinical AD as emerging cognitive biomarkers^3^. The parahippocampal gyrus (PHG) was reported as critical in spatial memory^4^ and demonstrates various effects in Alzheimer’s disease, including delay-dependent inaccuracy of memory-guided eye movements and poor long-term spatial memory^5,6^. A number of studies have suggested a link between the PHG and AD, with the potential for MRI-measured atrophy of the PHG functioning as a biomarker for preclinical AD^7–9^, while others propose that such cognitive impairments may not yet be present in cases of preclinical AD^10^.

**Fig. 1:**
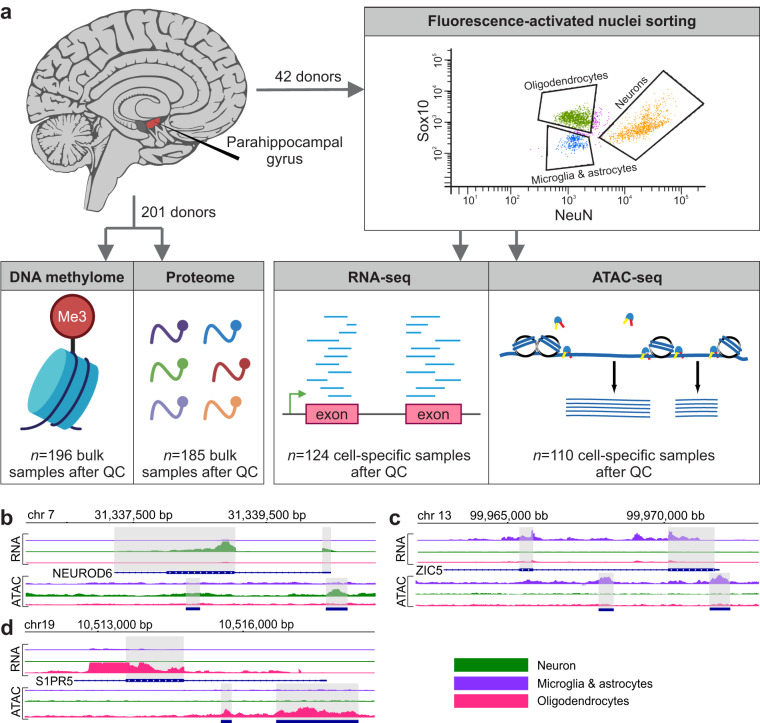
Study design and examples of transcriptome and epigenome landscape around three selected cell type markers. (**a**) Study design: Dissections from PHG brain region of AD case and control subjects were obtained from frozen human postmortem tissue. Nuclei were subjected to fluorescence-activated nuclear sorting to yield three cell populations, followed by RNA-seq and ATAC-seq profiling and subsequent downstream analyses to perform quality control and identify cell type-specific open chromatin regions. (**b**–**d**) Examples of cell-specific chromatin accessibility and gene expression for three cell type markers, i.e. (**b**) NEUROD6 (neurons), (**c**) ZIC5 (microglia & astrocytes) and (**d**) S1PR5 (oligodendrocytes).

Next-generation sequencing (NGS) is an example of non-clinical research that has enhanced our understanding of AD. Since the development of NGS, multiple genes implicated in AD risk and pathogenesis have been identified. Early NGS studies focused on performing deep resequencing of established early-onset AD genes, namely amyloid precursor protein (APP), presenilin 1 (PSEN1), and presenilin 2 (PSEN2), all extremely rare and accounting for less than 5% of cases^11^. More recent whole genome sequencing (WGS) studies focused on both rare and common risk variants for the more complex, and also more common, late-onset type of AD^12–15^. Due to the inherent difficulty in obtaining fresh specimens, most molecular studies of the human brain are restricted to frozen post-mortem samples. Working with frozen material is not without its challenges, including the loss of cytoplasm (and, with it, many cell-specific antigens) as a consequence of freeze-thawing. Nevertheless, an increasing number of human brain studies have employed cell-type specific nuclear markers to isolate nuclei of interest via Fluorescence-Activated Nuclear Sorting (FANS)^16–18^. In our recent study, FANS was utilized to isolate neuronal and non-neuronal samples (using an Anti-NeuN antibody) from AD cases and controls to identify cell-specific epigenetic changes associated with AD progression^19^. Here, we enlarged the panel of antibodies used by including SOX10, to further sort non-neuronal samples into oligodendrocytes (NeuN-/Sox10 + ) and microglia/astrocytes (NeuN-/Sox10-). In total, we have generated 124 cell-specific transcriptome samples (FANS-sorted RNA-seq), 110 cell-specific epigenome samples (FANS-sorted ATAC-seq) as well as 196 bulk DNA methylome samples and 185 bulk proteome samples. The newly generated data sets expand the panel of genomic assays in the Mount Sinai Brain Bank AD cohort (MSBB-AD)^20^ and increase cell-specific resolution (Fig. 1).

## Methods

### Cohort data collection

**Fig. 2:**
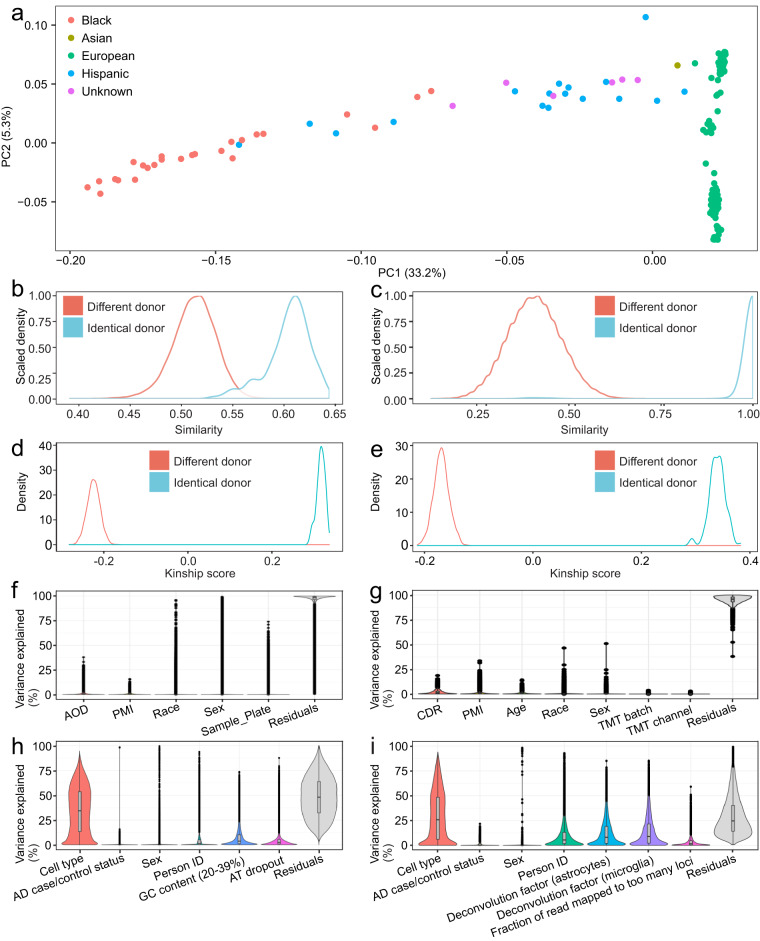
Analysis of cohort ancestry, genetic similarity and assay variance. (**a**) Top two principal components of per-sample genetic ancestry estimation. (**b**–**e**) Distribution of genetic similarities estimated between WGS samples and proteomics samples (**b**), DNA methylation samples (**c**), RNA-seq samples (**d**), ATAC-seq samples (**e**). Colors denote whether the sample pairs are originating from the same or different brain donors. (**f**–**i**) Variance explained by biological and technical covariates for DNA methylation sites (**f**), proteins (**g**), ATAC-seq genes (**h**) and RNA-seq peaks (**i**).

In this study, we generated a multi-omics data set from the frozen brain tissue of 201 subjects of predominantly European ancestry (Fig. 2a) obtained from the Mount Sinai NIH Neurobiobank^20^. All neuropsychological, diagnostic and autopsy protocols were approved by the Mount Sinai and JJ Peters VA Medical Center Institutional Review Boards^20^. Extensive cognitive and neuropathological assessment as well as demographic data were already available for all donors^21^. For bulk proteome and DNA methylome data, we generated the samples for all 201 donors. For RNA-seq transcriptome and ATAC-seq epigenome data, we selected only 42 donors (21 AD cases and 21 controls with either no discernable neuropathology or cognitive complaints) and performed FANS to generate 3 cell type specific samples per donor. The donors were selected to represent the full spectrum of clinical and pathological severity based on the following phenotypes: (1) case–control status defined using the Consortium to Establish a Registry for Alzheimer’s Disease (CERAD) criteria^22^, i.e. 1 = normal, 2 = definite AD, 3 = probable AD, and 4 = possible AD; (2) Braak AD-staging score for the progression of neurofibrillary neuropathology (Braak and Braak score^23,24^); (3) mean density of neuritic plaques (PlaqueMean); and (4) assessment of dementia based on the Clinical Dementia Rating scale (CDR)^25^.

### Bulk proteomics data

#### Tandem mass tag assays

The post-mortem brain samples were lysed in the fresh lysis buffer (50 mM HEPES, pH 8.5, 8 M urea, and 0.5% sodium deoxycholate) with the established protocol^26^. Protein concentrations of the lysates were measured by the BCA assay (Thermo Fisher Scientific) and further validated by short SDS Coomassie-stained gels^27^. Approximately 0.1 mg of quantified proteins in the lysis buffer with 8 M urea were digested in two steps: first with Lys-C (Wako, 1:100 w/w) at 21 °C for 2 h, and then with 4-fold dilution to reduce urea to 2 M followed by trypsin digestion (Promega, 1:50 w/w) at 21 °C overnight. The resulting peptide samples were acidified, desalted with Sep-Pak C18 cartridge (Waters), and then dried. These samples were re-dissolved in 50 mM HEPES (pH 8.5) for TMT reaction for 30 mins, and equally pooled. The pooled samples were desalted and fractionated by offline basic pH reverse phase LC (an XBridge C18 column of 3.5 μm particle size, 4.6 mm × 25 cm, Waters), and each collected fraction was then analyzed by the acidic pH reverse phase LC coupled with MS/MS analysis^28^. The fractions were analyzed sequentially on a C18 column (75 µm × 15–30 cm, 1.9 μm resin from Dr. Maisch GmbH, 65 °C to reduce backpressure) coupled with a Q Exactive HF Orbitrap mass spectrometer (Thermo Fisher Scientific). In mass spectrometer (MS) settings, positive ion mode and data-dependent acquisition were applied with one full MS scan followed by 20 MS/MS scans. MS1 scans were acquired at a resolution of 60,000, 1E6 AGC and 50 ms maximal ion time. After ion fragmentation with higher energy collision-induced dissociation (HCD, ~35% normalized collision energy and ~1.0 m/z isolation window with 0.3 m/z offset), MS2 spectra were acquired at a resolution of 60,000, fixed first mass of 120 m/z, 410–1600 m/z, 1E5 AGC, 100–150 ms maximal ion time, and ~15 sec of dynamic exclusion.

#### Computational processing

Tandem Mass Tag (TMT)-based proteomics analysis was utilized to profile protein expression abundance. Proteomics data analysis was performed as previously described^29^: the JUMP search engine^30^ was used to search MS/MS raw data against a composite target/decoy database^31^ to evaluate FDR. The protein database was generated by combining downloaded SwissProt, TrEMBL, and UCSC databases and removing redundancy (83,955 entries for human proteins), followed by concatenation with a decoy database. Major parameters included 15 ppm mass tolerance for precursor ions and 10 ppm for product ions, full trypticity, static modification of the TMT tags (+229.162932 Da) on Lys residues and peptide N termini and carbamidomethyl modification on cysteine (+57.02146 Da), dynamic modification for Met oxidation (+15.99492 Da), maximal miscleavage sites (*n* = 2), and maximal modification sites (*n* = 3). The resulting PSMs were filtered by precursor ion mass accuracy and minimal search score, and then grouped by peptide length, tryptic ends, modifications, miscleavage sites, and precursor ion charge state followed by the cutoffs of JUMP-based matching scores (Jscore and ΔJn) to reduce FDR below 1% for proteins. If one peptide could be generated from multiple homologous proteins, based on the rule of parsimony, the peptide was assigned to the canonical protein form in the manually curated SwissProt database. If no canonical form was defined, the peptide was assigned to the protein with the highest PSM number.

Proteins were quantified in the following steps, similar to previous reports^28,29,32^: (i) TMT reporter ion intensities of each PSM were extracted; (ii) the raw intensities were corrected according to isotopic distribution of each labeling reagent; (iii) PSMs with very low reporter ion intensities were excluded (e.g. minimum intensity < 1,000 and median intensity < 5,000); (iv) sample loading bias was corrected by normalization with the trimmed median intensity of all PSMs; (v) the mean-centered intensities across samples were calculated; (vi) protein relative intensities were summarized by averaging related PSMs; (vii) protein absolute intensities were derived by multiplying the relative intensities by the grand-mean intensity of the top three most highly abundant PSMs. In addition, we performed y1-ion based correction of TMT data^28^. Data QC based on protein quantification identified one batch (“batch 20”) as an outlier batch and was thus discarded. To generate a combined quantification table for multiple batches, a common sample (mixture of multiple samples) was included in each batch as an internal standard. MS intensities from different batches were normalized according to this internal standard.

### Bulk DNA methylation

#### Methylation array assays

Total genomic DNA was isolated from 10 mg of postmortem brain tissue dissected from the PHG region, using the Qiagen All Prep DNA/RNA Mini Kit, according to the manufacturer’s instructions (Qiagen, catalog# 80204). Tissues were first homogenized using Qiagen’s TissueLyzer LT (Qiagen, catalog# 69980) combined with 5 mm stainless steel beads (Qiagen, catalog# 69989). Next, the lysed tissues were loaded onto QIAmp spin columns to wash off any impurities. Purified DNA was eluted off from the columns using a low salt buffer. 200–500 ng DNA per sample were used for bisulfite conversion using the EZ-96 DNA Methylation-Lightning Kit (Zymo, catalog# D5033). Next, DNA samples were fragmented and hybridized to Infinium MethylEPIC BeadChips^33^ (Illumina, catalog# WG-317-1001). Lastly, hybridization signals were obtained through the Illumina iScan microarray scanner (Illumina, catalog# SY-202-1001).

#### Methylation data preprocessing

We streamlined a workflow to preprocess, normalize, and quality check (QC) Illumina 850 K methylation array data. We essentially followed the pipeline as described by Maksimovic *et al*.^34^. In brief, the R package “minfi” was utilized to preprocess and normalize the raw array data in IDAT format. We utilized the functions “read.metharray.sheet” and “read.metharray.exp” to import the sample metadata and the raw methylomic data into R, respectively, and the function “preprocessQuantile” for data normalization, and the functions “getBeta” and “getM” to calculate and output both β and M values of the 866,029 probes on the platform, respectively. For further quality control, we discarded CpG probes that were either internal control, or with low quality (detection p-value < 0.05)^34^, or known to overlap with common SNPs at the same CpG sites^34^. The genic annotation of the CpG sites was obtained from the annotation package for Illumina’s EPIC methylation arrays, i.e., IlluminaHumanMethylationEPICanno.ilm10b2.hg19. After removing 5 potentially mismatched samples, as described in the Technical Validation section, the β and M values of the QCed CpG probes were corrected for co-variables including batch.

### Cell-type-specific RNA sequencing

#### Fluorescence-activated nuclei sorting

From each dissection, 250 mg of frozen brain tissue was homogenized in a cold lysis buffer (0.32 M Sucrose, 5 mM CaCl2, 3 mM Magnesium acetate, 0.1 mM, EDTA, 10 mM Tris-HCl, pH8, 1 mM DTT, 0.1% Triton X-100) and filtered through a 40 µm cell strainer. The flow-through was underlaid with sucrose solution (1.8 M Sucrose, 3 mM Magnesium acetate, 1 mM DTT, 10 mM Tris-HCl, pH 8) and subjected to ultracentrifugation at 24,000 rpm for 1 hour at 4 °C. Pellets were resuspended in 500 µl DPBS and incubated in BSA (final concentration 0.1%) and anti-NeuN antibody (1:600, Alexa 647 conjugated, abcam Cat #ab190565) and anti-SOX10 (1:300, Alexa488 conjugated, R&D Systems Cat #IC28642G). Prior to FANS sorting, DAPI (Thermoscientific) was added to a final concentration of 1 µg/ml. Neuronal (DAPI + NeuN + SOX10-), oligodendrocytes (DAPI + NeuN- SOX10 + ) and microglia/astrocytes (DAPI + NeuN- SOX10-) nuclei were sorted into individual tubes (pre-coated with 5% BSA) using a FACSAria flow cytometer (BD Biosciences).

#### Library preparation and sequencing

For RNA-seq, nuclei were sorted into 1.5 ml low-bind microfuge tubes containing Extraction buffer, a component of the PicoPure RNA Extraction kit (Arcturus, Ca t# KIT0204). RNA was isolated in accordance with the PicoPure RNA Isolation kit’s manufacturer’s instructions. This included an RNase-free DNase treatment step (Qiagen, Cat # 79254). Samples were eluted in RNase-free water and stored at −80 °C until preparation of RNA-Sequencing libraries using the SMARTer Stranded Total RNA-Seq Pico Kit v1 or v2 (Takara Clontech Laboratories, Cat # 635005 or 634414, respectively), according to the manufacturer’s instructions. Following the construction of the RNA-seq libraries, libraries were analyzed on a TapeStation using a High Sensitivity D1000 ScreenTape (Agilent, Cat # 5067-5584) and quantification of the libraries was performed using the KAPA Library Quantification Kit. Libraries that passed QC were sequenced on Hi-Seq2500 (Illumina) obtaining 2 × 50 paired-end reads.

#### Computational processing

The raw reads were trimmed with Trimmomatic (v0.36)^35^ and then mapped to human reference genome hg38 using STAR (v2.5.3a)^36^. Following read alignment, expression quantification was performed at the transcript isoform level using RSEM (v1.3.0)^37^ and then summarized at the gene level. Gene quantifications correspond to GENCODE (v30)^38^. Gene count matrix was normalized by the trimmed mean of M-values (TMM)^39^ and filtered to keep only genes with over 1 count per million in at least 30% of the samples. RNA-SeqQC (v1.1.7)^40^ and Picard (v2.2.4) were used to generate quality control metrics. Quality control processes (described in Technical validation) removed 2 samples, resulting in a final count matrix of 124 samples by 21,383 genes. To correct for unwanted technical variance, we applied the step-wise covariate analysis based on the Bayesian information criterion (BIC)^41^. As a starting point for this analysis, a base model was chosen with the variables “cell_type by diagnosis_status” and “sex”. Then, it was tested, for each additional covariate, how many genes showed an improved BIC score minus how many showed a worse BIC score when the covariate was included in the linear regression model compared to when it wasn’t. A covariate was then required to improve the mean BIC per gene by at least 5 for it to be included in the final model. This model selected 3 covariates: “reads_mapped_to_too_many_loci” (i.e. fraction of discarded reads by STAR aligner; this serves as a proxy to the technical quality of the sample) and two deconvolution metrics, i.e. predicted proportion of microglia and astrocytes in each sample, thus compensating for limitations of our experimental design that sorted cells from microglia and astrocytes together. The effect of those three technical covariates was regressed out to generate the normalized count matrices.

### Cell-type-specific ATAC sequencing

#### Generation of ATAC-seq libraries and sequencing

ATAC-seq libraries were generated from cell-sorted brain tissue dissection (see the section “Fluorescence Activated Nuclei Sorting” for RNA-seq) using an established protocol^42^ with minor modifications. In brief, 100,000 sorted nuclei were centrifuged at 500 g for 10 min at 4 °C. Pellets were resuspended in transposase reaction mix (25 μL 2x TD Buffer (Illumina Cat # FC-121-1030) 2.5 μL Tn5 Transposase (Illumina Cat # FC-121-1030) and 22.5 μL Nuclease Free H_2_O) on ice. Reactions were incubated at 37 °C for 30 min and then purified using the MinElute Reaction Cleanup kit (Qiagen Cat # 28204), eluting in 10 µL of buffer EB. Following purification, library fragments were amplified using the Nextera index kit (Illumina Cat # FC-121-1011) under the following cycling conditions: 72 °C for 5 minutes, 98 °C for 30 seconds, followed by thermocycling at 98 °C for 10 seconds, 63 °C for 30 seconds, and 72 °C for 1 minute for a total of 5 cycles. To prevent saturation due to over-amplification, a 5 µl aliquot was then removed and subjected to qPCR for 20 cycles to calculate the optimal number of cycles needed for the remaining 45 μL reaction. The additional number of cycles was determined by first plotting linear Rn vs. Cycle and secondly calculating the cycle number corresponding to 1⁄4 of maximum fluorescence intensity. In general, adding 4-6 cycles to this estimate was found to yield optimal libraries, as determined by analysis on Tapestation D5000 ScreenTapes (Agilent technologies Cat # 5067-5588). Libraries were then resolved on 2% agarose gels and fragments ranging in size from 100-1000 bp were excised and purified (Qiagen Minelute Gel Extraction Kit – Qiagen Cat # 28604). Prior to sequencing, libraries were quantified with the Qubit dsDNA HS assay kit (Invitrogen Cat # Q32851) and by quantitative PCR (KAPA Biosystems Ca # KK4873), and fragment sizes estimated using Tapestation D5000 ScreenTapes (Agilent technologies Cat # 5067-5588). Libraries that passed QC were normalized for concentration and sequenced on Hi-Seq2500 (Illumina), obtaining 2 × 50 paired-end reads.

#### Computational processing

Similar to RNA-seq processing, trimming of low-quality base pairs and adapter sequences was performed by Trimmomatic (v0.36)^35^. Then, reads were mapped to hg38 by STAR (v2.7.0)^36^. Reads mapped to multiple loci, mitochondrial genome or duplicate reads were removed by samtools (v0.1.19)^43^. To increase the sequencing depth for peak calling, all samples were downsampled to the same size and, then, merged into three separate BAM files by their cell type identity. Cell-specific peaks were called by MACS2^44^ and merged into a final consensus of 263,265 peaks. Peaks overlapping ENCODE blacklisted regions^45^ were removed. The peak count matrix was normalized by the trimmed mean of M-values (TMM)^39^ and filtered to keep only peaks with over 1 count per million in at least 20% of the samples. Picard (v2.2.4) and phantompeakqualtools (v2.0) were used to generate quality control metrics. Quality control processes (described in Technical validation) removed 16 samples, resulting in a final count matrix of 110 samples by 257,336 peaks. Then, we applied the same covariate selection model utilizing repeated BIC model as for RNA-seq, This model selected two covariates: “GC_coverage_20-39” (i.e., normalized read coverage over each quintile of GC content ranging from 20 – 39%) and “AT_dropout” that improved a net of 68.5% and 21.5% of peaks. The effect of those two technical covariates was regressed out to generate the normalized count matrices.

## Data Records

All data described herein are available for use by the research community and have been deposited in the AMP-AD Knowledge Portal in study-specific folder^46^. These include sample metadata^21^, as well as raw and processed sequencing data for ATAC-seq, RNA-seq, proteome and DNA methylation^46^.

## Technical Validation

### Bulk proteomics data quality control

We performed sample alignment between proteomics data and matched WGS^47^ data from the same cohort using two different strategies. In the first strategy, we utilized a proteogenomics approach to first identify sample-specific peptides with mutations, followed by proteogenomics-based genotype inference and sample alignment using the SMAP software^48^. Briefly, by constructing a customized protein database using SNVs detected from WGS data, peptides with sample-specific mutations were identified using the JUMPg software^49^. The resulting peptides were quantified and processed by SMAP for sample alignment with two steps: (i) inference of sample-specific genotype based on TMT-based quantification while taking the genotype dosage information in the WGS data as prior knowledge; and (ii) sample verification and correction by comparing the inferred genotypes versus the mutation profiles of the matched WGS sample. Five proteomics samples were identified to be potentially mislabeled. In the second strategy, we utilized the software MODMatcher^50^. Briefly, the normalized proteomics data were corrected for TMT batch using a random effect regression model by R package variancePartition^51^, and subsequently corrected for covariates including PMI, age, race, and sex using linear regression (Fig. 2g). Then protein quantitative trait loci (pQTLs) were computed with R package MatrixEQTL^52^ by integrating covariates-corrected proteomics data with the WGS data. Genotypes at the most significant cis-pQTL of each cis-pQTL bearing protein were imputed from the protein expression data using an algorithm developed in the software MODMatcher^50^. Genotype consistency was computed for all possible sample pairs between the imputed genotype data from proteomics and the observed genotype data from WGS (Fig. 2b). Following MODMatcher^50^, a proteomics sample was considered self-aligned with the corresponding WGS sample if its same-donor WGS sample was among the top 3 matches ranked by the genotype consistency score. Meanwhile, best-matched proteomics samples for each WGS sample were also identified based on the genotype consistency score. As a result, 183 proteomics samples were self-aligned. Among the 7 proteomics samples that were not self-aligned with WGS, 4 were considered potentially mislabeled as each showed a reciprocal best match with a WGS sample from a different donor. Notably, all these 4 proteomics samples were among the mislabeled samples detected by JUMPg. Therefore, we corrected the donor identifiers for the 4 mislabeled samples and discarded the remaining problematic samples (total 4) detected by either JUMPg or MODMatcher. One mislabeled sample became a duplication after label correction and hence was discarded as well. Lastly, the retained normalized proteomics data (*n* = 185) with properly matched WGS data were corrected for covariates including TMT batch, PMI, age, race, and sex.

### Bulk DNA methylation data quality control

To assure the high quality of the DNA methylation data, we first evaluated if the DNA methylation samples can be properly aligned to their corresponding WGS samples. For this purpose, we carried out the genotype inference on 59 control probes querying high-frequency SNPs by Illumina’s EPIC chip. Following a prior practice^53^, a mixed model assuming distinct hybridization signal distribution for different genotypes was trained to predict sample genotypes for each of these probes. Subsequently, a genotype concordance score was computed by comparing the inferred genotypes with the WGS-based genotypes. While the majority of the DNA methylation samples showed a high genetic concordance with their corresponding WGS samples (genotype similarity score close to 1), 5 methylation samples exhibited a low genotype concordance with their respective WGS counterparts (genotype concordance score < 0.9) and were hence labeled as mismatched samples and discarded from the analysis (Fig. 2c).

### RNA-seq quality control

**Fig. 3:**
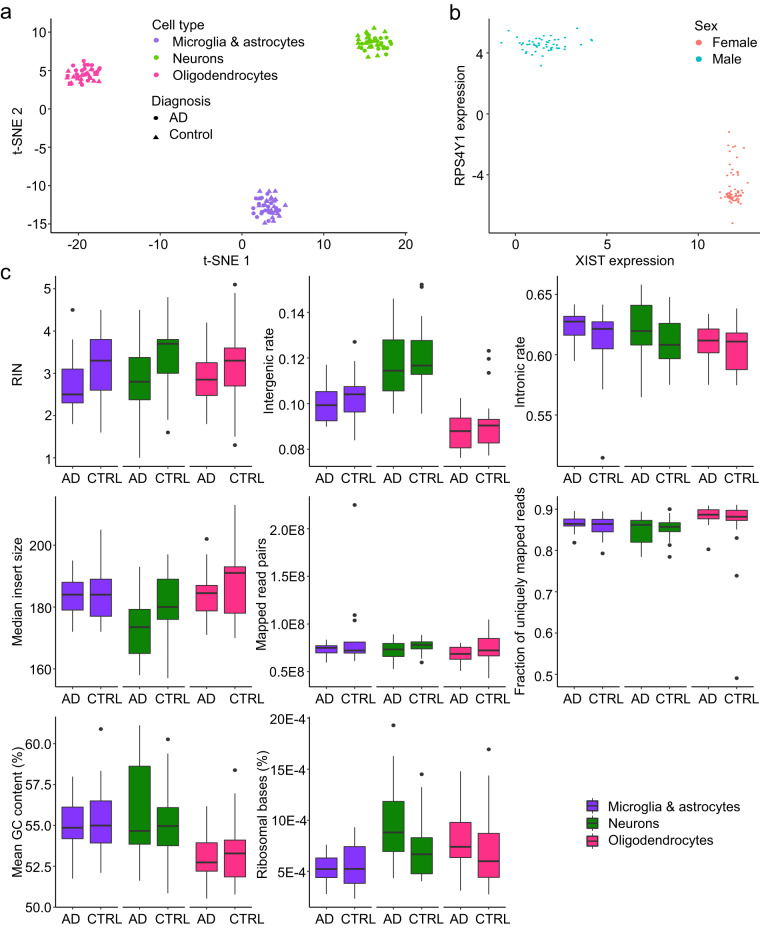
Analysis of RNA-seq dataset. Diagnosis groups are defined by CERAD metrics, i.e. AD: CERAD = (2-4), CTRL: CERAD = 1. **(a)** t-SNE clustering. **(b)** Sex check based on quantification of the expression of male- (RPS4Y1) and female-specific genes (XIST). **(c)** RNA-seq quality control metrics stratified by disease status and cell subtype: RNA integrity number (RIN), intergenic rate, intronic rate, median insert size, counts of mapped read pairs, percentage of uniquely mapped reads, mean GC content and percentage of ribosomal bases. t-test comparison on the distributions of values of AD cases and controls for all QC metrics in three cell types revealed that only 2 metrics in 2 cell types are different before correction for multiple testing but not after FDR correction, i.e. the percentage of ribosomal bases in neurons (p-value = 0.030, FDR-corrected q-value = 0.090) and the number of mapped reads in oligodendrocytes (p-value = 0.048, FDR-corrected q-value = 0.144). Box plots are centered on median, bounds defined between the 25th and 75th percentile with minimum and maximum defined as median ± 1.5 × interquartile range, and whiskers extending to the lowest/highest value within this range.

A total of 126 RNA-seq samples were integrated into a single analysis across all cell types and AD case/control status in order to perform joint quality control. Dimensionality reduction techniques calculated on gene count matrix were used to confirm the successful clustering of samples by cell types, with the exception of two outlying samples that were excluded (laboratory notes indicated that those two samples yielded very low concentrations of RNA, indicative of low tissue quality) (Fig. 3a; removed samples not shown). The remaining 124 samples had acceptable values for the following RNA-seq quality control metrics: RNA integrity number (RIN) (mean 3.1, sd ± 0.84), intergenic rate (mean 10.3%, sd ± 1.6%), intronic rate (mean 61.4%, sd ± 2.2%), ribosomal RNA rate (mean 0.07%, sd ± 0.03%), mapped read pairs (mean 75 × 10^6^, sd ± 1.7 × 10^6^), percentage of uniquely mapped reads (mean 86.1%, sd ± 4.5%), median insert size (mean 182 bp, sd ± 10 bp) and mean GC content (mean 54.6%, sd ± 2.3%) (Fig. 3c). In order to confirm that the expression of genes on sex chromosomes is consistent with the reported sex, RPS4Y1 and XIST were selected as representatives of sex-specific genes, and all samples showed distinct clustering by reported sex (Fig. 3b). To verify donor identity of all samples, we used kinship coefficient from KING v1.913^54^ to compare the per-sample variants called from raw sequencing reads to the variants from existing WGS reference^47^. All detected swaps between samples were corrected by sample re-labeling, however, two potentially contaminated samples with low similarity to all genotypes, including the expected genotype, were excluded. After performing all steps of genotype concordance analysis, we observed clear and unambiguous separation of *n* = 124 samples from 21 donors (Fig. 2d).

### ATAC-seq quality control

**Fig. 4:**
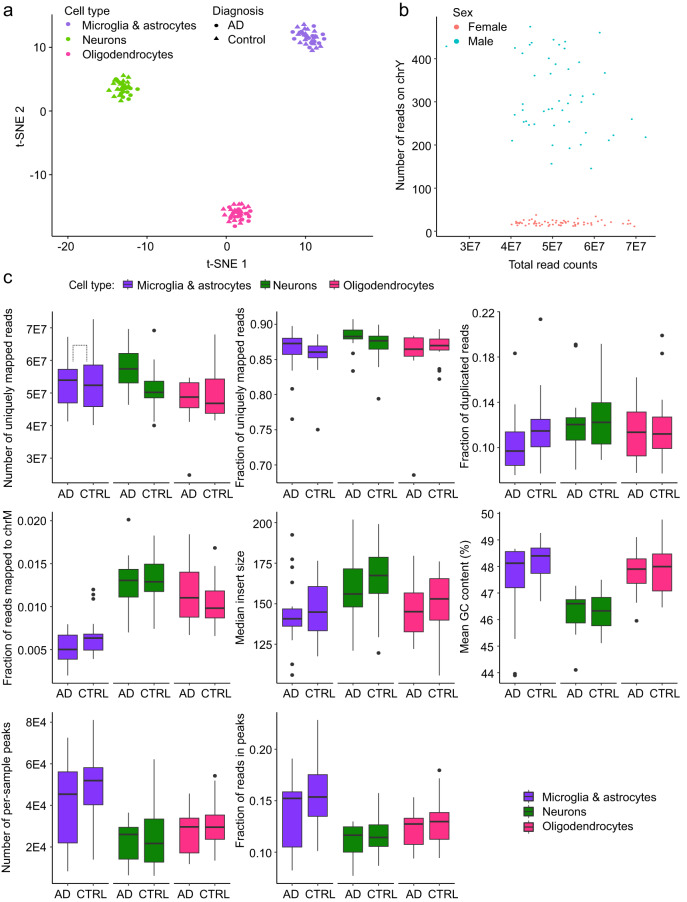
Analysis of ATAC-seq dataset. Diagnosis groups are defined by CERAD metrics, i.e. AD: CERAD = (2-4), CTRL: CERAD = 1. **(a)** t-SNE clustering. **(b)** Sex check based on quantification of the number of reads on chromosome Y (outside the pseudoautosomal region). **(c)** ATAC-seq quality control metrics stratified by cell subtype and AD disease status: counts of uniquely mapped reads, fraction of uniquely mapped reads, fraction of duplicated reads, fraction of reads mapping to the mitochondrial genome, median insert size, mean GC content, number of per-sample peaks and FRiP (fraction of reads in peaks). t-test comparison on the distributions of values of AD cases and controls for all QC metrics in three cell types revealed that only 1 metrics in 1 cell type is different before correction for multiple testing (the fraction of reads mapped on chrM: p-value = 0.041, FDR-corrected q-value = 0.123) and 1 metrics in 1 cell type is statistically significantly different after correction for multiple testing. (the number of uniquely mapped reads in neurons: p-value 0.008, FDR-corrected q-value = 0.234). Box plots are centered on median, bounds defined between the 25th and 75th percentile with minimum and maximum defined as median ± 1.5 × interquartile range, and whiskers extending to the lowest/highest value within this range.

Similar to RNA-seq quality control, we performed a joint analysis of all 122 sequenced ATAC-seq samples to detect outlying and low-quality samples. All samples passed our QC metrics criteria for minimum mappability (more than 50% required), minimum fraction of reads in peaks (more than 4% required) and maximum fraction of reads mapped to the mitochondrial genome (less than 3% required). However, 6 samples were removed due to the low signal-to-noise ratio as we required more than 3,000 narrow peaks per sample. An additional 6 samples were removed due to low cell type specific signal detected by clustering analysis and visually confirmed in IGV by looking at open chromatin accessibility signal within promoters of cell-specific genes. After completion of QC steps, the remaining samples showed clear cell type separation (Fig. 4a). The remaining 110 samples had acceptable values for the following ATAC-seq quality control metrics: number of narrow peaks called per sample (mean 32,906, sd ± 18,170), the fraction of reads in peaks (mean 13%, sd ± 2.9%), the fraction of reads mapping to the mitochondrial genome (mean 0.98%, sd ± 0.4%), median insert size (mean 116 bp, sd ± 24 bp), the fraction of reads that were uniquely mapped (mean 0.865, sd ± 0.029), mean GC content (mean 46.4%, sd ± 1.2%), the number of uniquely mapped reads (mean 59 × 10^6^, sd ± 9.3 × 10^6^) and the fraction of duplicated reads (mean 0.117, sd ± 0.029%) (Fig. 4c). We also carried out sex check by comparing per-sample numbers of all mapped reads versus chromosome Y reads, confirming distinct clustering by reported sex (Fig. 4b). Lastly, we checked the identity of a final set of *n* = 110 samples from 21 donors using the same approach as explained for RNA-seq data and corrected all swaps and mislabelings (Fig. 2e).

## Usage Notes

**Fig. 5:**
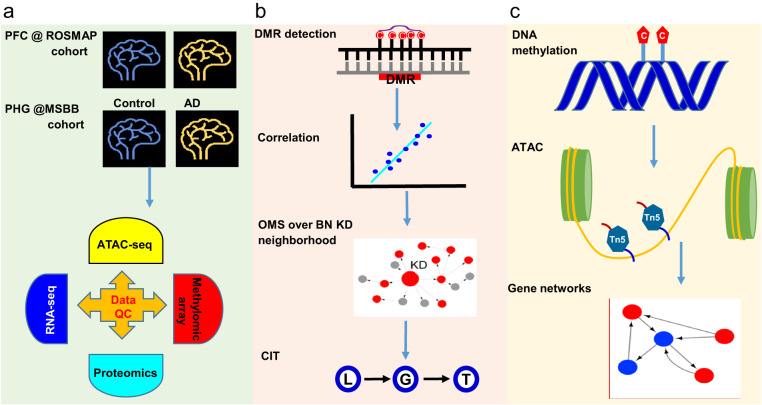
Integration of large-scale multi-omics data in AD to develop predictive molecular network models for identifying key driving factors of AD. (**a**) Overview of the study design, multi-omics data collection and quality check. (**b**) DNA methylation regulates gene and protein expression as well as their coexpression networks. DMRs were first identified, followed by correlation/association analyses of DMRs with AD clinical traits, network module relevance to AD, gene/protein network connectivity, AD risk genes, and Aβ gene signatures. Overall methylation score (OMS) was calculated to model the net effects of DMRs over gene expression, and was further investigated for their relevance to network metrics; Last, the causal relationship of methylation and ATAC domain to gene expression was evaluated by cit (causal inference test). BN: Bayesian network, KD: key driver; OMS: overall methylation score, DMR: differentially methylated region, CIT: causal inference test. (**c**) Methylomic regulatory cascade. The results suggest that DMRs are likely causal to ATAC domain activity in regulating the expression of genes in the networks.

As a usage example, here we summarize the analytic flow and the key findings from our recent publication^55^ in which we integrated the multi-omics data in the MSBB-AD cohort which was developed through a previous study^55^ and this current study (termed as the discovery cohort) and the Religious Orders Study and Memory and Rush Aging Project^56,57^ (ROSMAP) cohort with multiomics data from the dorsolateral prefrontal cortex (the validation cohort). The discovery cohort includes matched epigenomic (ATAC-seq), methylomic, transcriptomic (RNA-seq) and proteomic data from the PHG, as described in the previous sections in this paper, while the validation cohort includes methylomic, transcriptomic (RNA-seq) and proteomic data, along with ATAC-seq data and H3K9ac domain atlas in the prefrontal cortex (PFC) region^58^ (Fig. 5a).

As shown in Fig. 5b, we first identified AD-associated methylomic changes by computing differentially methylated probes and differentially methylated regions (DMRs). In the MSBB AD, 270 DMRs were found to be not only associated with AD clinical and pathological traits cohort and the expression levels of many genes and proteins differentially expressed between AD and controls but also enriched for known AD GWAS risk genes and the Aβ pathways. To model and quantify the overall effect of DNA methylation on individual genes and proteins, we developed a novel statistic, termed overall methylation score (OMS)^55^, and revealed that in the gene or protein co-expression network modules which were most strongly associated with AD, their member genes or proteins generally had a high amplitude of OMS that was also correlated with the respective gene/protein expression changes between AD and controls. We also found that, in the Bayesian causal networks, the top-ranked key drivers tended to be regulated by methylation. Finally, to investigate the causal relationship between DMRs and ATAC peaks on gene expression, the causal inference test (CIT)^59^ was performed on DMRs, ATAC peaks, and associated genes or proteins. Our analysis identified thousands of significant causal chains with a relationship of DMR → ATAC → gene/protein, but none of the relationship of ATAC → DMR → gene/protein, suggesting that DMRs likely influenced gene/protein expression via ATAC peak domains in AD, rather than ATAC peak domains influenced gene/protein expression via DNA methylation. In summary, our integrative analysis of the multi-omics data reveals a detailed signaling map of the regulatory cascade among DNA methylation, epigenomic chromatin accessibility, transcription and translation in AD (Fig. 5).

## Data Availability

The source code demonstrating the work with the dataset is available at 10.5281/zenodo.7818443^60^.
